# High prevalence but limited evidence in complementary and alternative medicine: guidelines for future research

**DOI:** 10.1186/1472-6882-14-46

**Published:** 2014-02-06

**Authors:** Felix H Fischer, George Lewith, Claudia M Witt, Klaus Linde, Klaus von Ammon, Francesco Cardini, Torkel Falkenberg, Vinjar Fønnebø, Helle Johannessen, Bettina Reiter, Bernhard Uehleke, Wolfgang Weidenhammer, Benno Brinkhaus

**Affiliations:** 1 Institute for Social Medicine, Epidemiology, and Health Economics, Charité Universitätsmedizin, Luisenstr. 57, 10098 Berlin, Germany; 2 Complementary and Integrated Medicine Research Unit, University of Southampton, Southampton, UK; 3 Center for Integrative Medicine, School of Medicine, University of Maryland, Baltimore, USA; 4 Institute of General Practice, Klinikum rechts der Isar, Technische Universität München, Munich, Germany; 5 Institute of Complementary Medicine (KIKOM), University of Bern, Bern, Switzerland; 6 Healthcare and Social Agency of Emilia Romagna Region, Bologna, Italy; 7 Research Unit for Integrative Healthcare Research, Karolinska Institute, Stockholm, Sweden; 8 I C – The Integrative Care Science Center, Järna, Sweden; 9 National Research Center on Complementary and Alternative Medicine (NAFKAM), University of Tromsø, Tromsø, Norway; 10 Institute of Public Health, Research Unit Health, Man and Society, University of Southern Denmark, Odense, Denmark; 11 International Academy for Holistic Medicine, Vienna, Austria; 12 Institute of Complementary Medicine, Department of Internal Medicine, University Hospital Zurich, Zurich, Switzerland; 13 Competence Centre for Complementary Medicine and Naturopathy, Klinikum rechts der Isar, Technische Universität, Munich, Germany

**Keywords:** Complementary and alternative medicine, Research strategy, Randomized clinical trials, Safety, Qualitative studies, Comparative effectiveness research

## Abstract

The use of complementary and alternative Medicine (CAM) has increased over the past two decades in Europe. Nonetheless, research investigating the evidence to support its use remains limited. The CAMbrella project funded by the European Commission aimed to develop a strategic research agenda starting by systematically evaluating the state of CAM in the EU. CAMbrella involved 9 work packages covering issues such as the definition of CAM; its legal status, provision and use in the EU; and a synthesis of international research perspectives. Based on the work package reports, we developed a strategic and methodologically robust research roadmap based on expert workshops, a systematic Delphi-based process and a final consensus conference. The CAMbrella project suggests six core areas for research to examine the potential contribution of CAM to the health care challenges faced by the EU. These areas include evaluating the prevalence of CAM use in Europe; the EU cititzens’ needs and attitudes regarding CAM; the safety of CAM; the comparative effectiveness of CAM; the effects of meaning and context on CAM outcomes; and different models for integrating CAM into existing health care systems. CAM research should use methods generally accepted in the evaluation of health services, including comparative effectiveness studies and mixed-methods designs. A research strategy is urgently needed, ideally led by a European CAM coordinating research office dedicated to fostering systematic communication between EU governments, the public, charitable and industry funders, researchers and other stakeholders. A European Centre for CAM should also be established to monitor and further a coordinated research strategy with sufficient funds to commission and promote high quality, independent research focusing on the public’s health needs and pan-European collaboration. There is a disparity between highly prevalent use of CAM in Europe and solid knowledge about it. A strategic approach on CAM research should be established to investigate the identified gaps of knowledge and to address upcoming health care challenges.

## Background

Complementary and alternative medicine (CAM) in the European Union (EU) includes practices such as acupuncture, anthroposophic medicine, aromatherapy, herbal medicine, homeopathy, kinesiology, massage, naturopathy, shiatsu, traditional Chinese medicine, and yoga. Over the last 25 years, the use of CAM has risen in Western industrialized countries [1-4] and CAM has been used often by patients with chronic conditions, such as cancer or chronic pain, or in situations where conventional treatment options have been limited [5].

The new EU framework programme ‘Horizon 2020’ emphasizes the consequences of demographic change in the EU, focusing in particular on elderly patients, who often experience multiple, chronic conditions. CAM may therefore have an important role to play in improving health and well-being in an ageing EU population, including the management of chronic conditions, the prevention of illness, and the promotion of health. Due to substantial gaps in knowledge about CAM, the precise role of CAM in these areas remains unclear.

**Figure 1:**
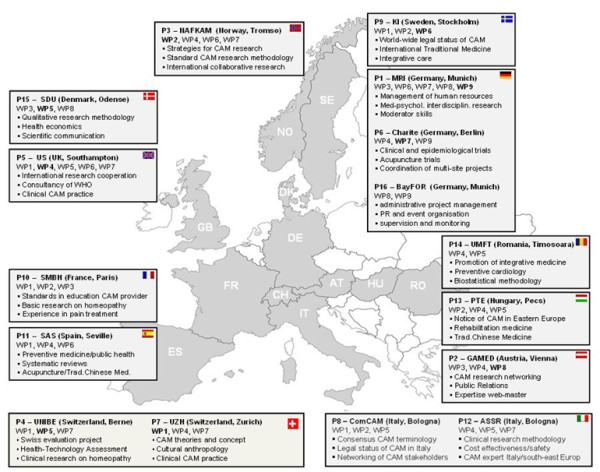
The CAMbrella Consortium funded by Framework Program 7.

In 2009 the European Commission called for coordinated and collaborative efforts to assess the current state of CAM in Europe in terms of definition, legal status, prevalence, provision and the attitudes of EU citizens as well as to identify the best methodological and strategic approaches to research in this field. The coordination project CAMbrella included 16 institutions from 12 European countries (Figure 1) and was funded by the 7th Framework Programme to address these issues in a systematic manner [6].

Based on the findings of this assessment, a roadmap was developed to point out which areas of CAM research appear promising, which methods should be applied, and how the EU could foster a more coordinated approach. Our suggestions are limited to the field of clinical and epidemiological research. Basic research - although also of great relevance - is not addressed within CAMbrella. The recommendations made as part of the roadmap are based on the knowledge and experience of European and international researchers in the field, and are designed to improve the quality and methodology of CAM research, as well as its relevance to clinical decision making.

The aim of this article is to present the CAMbrella research roadmap based on the key results of the main CAMbrella work packages to a wider audience of scientists, health care providers, decision makers and the public.

## Methods

**Figure 2:**
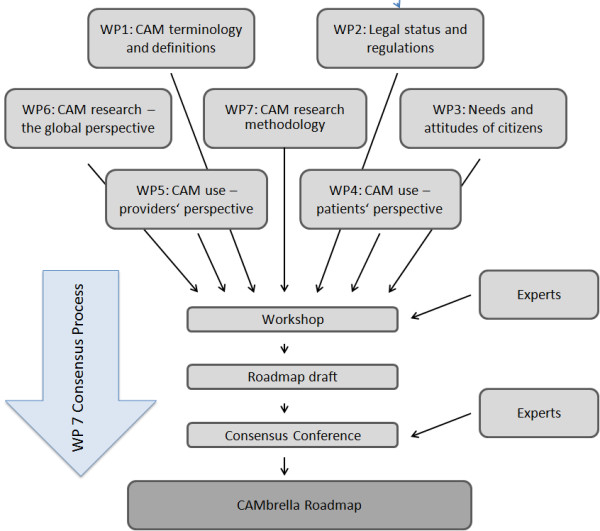
Roadmap development process.

The CAMbrella roadmap includes strategic and methodological recommendations for future CAM research and was developed systematically over three years (2010–12) (Figure 2). Within each CAMbrella work package, we used different methods to answer the questions raised, including systematic reviews, qualitative data analysis, workshops, focus groups, qualitative data synthesis, Delphi consensus techniques, and expert conferences. In particular, the CAMbrella group conducted four systematic reviews to describe the current state of legislation pertaining to CAM in the EU [7], citizens’ and patients’ attitudes and needs regarding CAM [8], the prevalence of CAM use [9], and key issues in clinical and epidemiological research on CAM [10]. Furthermore, CAM definitions and terminology were analysed and a pragmatic definition of CAM was developed [11]. We also evaluated the current state of CAM provision in the EU [12]. Lastly, international perspectives on CAM research were assessed as part of a qualitative study that included experts and organizations from Asia, Australia, Europe and North America [13] (Table 1).

**Table 1:** Work packages within the CAMbrella project

**Work package**	**Lead institution**	**Goal**	**Methods**	**Reference**
WP1	University of Zurich	Definition of CAM	Expert consensus panel	[11]
WP2	University of Tromsø	Legal status	Systematic review of legislative documents	[7]
WP3	University of Southern Denmark, Odense	Citizens’ perspective	Systematic review of scientific literature	[8]
WP4	University of Southampton	Patients’ perspective	Systematic review of scientific literature	[9]
WP5	University of Bern	Providers’ perspective	Review of scientific literature/grey literature and personal communication with key stakeholders	[12]
WP6	Karolinska Institutet, Stockholm	International perspective	Interviews with key international institutions	[13]
WP7	Charité University Medical Center, Berlin	Research methods	Systematic review of scientific literature	[10]
WP8	International Academy for Holistic Medicine, Vienna	Dissemination and communication	-	[15]
WP9	Technische Universität München	Project coordination	-	[6]

These studies served as the basis for a three-day expert workshop on CAM research strategies and methods, which took place in Castellaro, Italy, from September 7-9, 2011. A draft of the recommendations was developed and finalized in two written Delphi rounds between January and March 2012. The final draft was sent to all members of the CAMbrella consortium and to the CAMbrella advisory board in April 2012 for feedback. A revised version was presented at a one-day consensus conference, which took place in Järna, Sweden, on May 10, 2012. The roadmap was revised following the suggestions of the participants in the consensus conference and ultimately approved by CAMbrella’s Scientific Steering Committee in September 2012.

A total of 36 experts participated in the process, including all members of the CAMbrella consortium (n = 24, from 12 different European countries) (Figure 1); the CAMbrella Advisory Board (n = 7, from 5 European countries), which consisted of CAM doctors, practitioners, patient advocates and manufacturers; and 5 external, internationally recognized experts on CAM research methodology from the USA (1), the UK (2) and Germany (2).

## Findings

The CAMbrella vision for the year 2020 is that research into CAM will provide a broad but relevant and comprehensive evidence base, enabling the EU public and health care providers to make informed decisions about CAM use, both for individuals and for society as a whole.

A main issue is to define clinical situations in which CAM treatment is appropriate. The CAMbrella roadmap puts forward a research agenda for CAM that is designed to address future health care challenges in the EU. In particular, these include the increasing prevalence of chronic conditions and ongoing financial constraints.

To achieve this goal, concerted efforts are needed. The CAMbrella roadmap provides an outline for quantitative and qualitative research, suggesting six core areas covering important gaps in knowledge (Table 2). Hence, the roadmap also suggests a methodological framework for future CAM research based on the experiences of the past and makes a range of recommendations on steps the EU could take to foster these efforts.

**Table 2:** Overview of key research in the CAMbrella research roadmap

**Key research areas**	**Aim**	**Recommendation**	**Research methods**	**Specifics**
**CAM prevalence**	To obtain valid, comparable and comprehensive data on prevalence of CAM use	Structured EU-wide approach	Cross-sectional studies	Using standard definitions, develop standardized questionnaires for surveys in European languages
**Needs and attitudes of citizens and providers**	To address issues that are relevant to the EU public	Obtain data on how the diversity of the EU influences attitudes about CAM	Cross-sectional studies; qualitative interview studies	Involve the public as stakeholders in project development
**CAM safety**	To allow the risks of CAM to be estimated	Address safety in studies and establish an EU-wide monitoring system	Observational studies; clinical studies; single case studies	Clarify safety terminology; address safety in CAM studies where appropriate
**Comparative effectiveness research**	To support clinical and health care policy decision making with suitable research data	Future research should primarily investigate CAM in real-world settings	Comparative effectiveness research, including pragmatic clinical trials	Compare meaningful alternatives; include health economic evaluations
**Effects of context and meaning**	Understand the extent to which the clinical effects of CAM are due to non-specific treatment effects	Assess the nature, size and influence of potential non-specific treatment effects	Mixed methods (qualitative studies within clinical trials)	Research question is not specific to CAM, but of general interest
**Models of CAM integration**	To investigate different models of CAM integration	Describe, evaluate and further develop models for CAM integration	Mixed methods	Include the public’s view on models of CAM integration

### Research into the prevalence of CAM use

Our systematic review of the prevalence of CAM use in the EU concluded that the included studies were too heterogeneous in terms of definitions and methodological rigor to draw even a tentative picture [9]. The lifetime prevalence of CAM use ranged from 0.3% to 86% in the populations studied, and data for more than half of the EU member states were lacking entirely. Data from our evaluation of CAM provision suggest that there may be roughly 300,000 providers of CAM in its various forms practicing within the EU, of whom approximately 60% are non-medical providers [12].

A clear picture of CAM use is crucial for providers, purchasers and health policy makers alike. We recommend that the prevalence of CAM use in the EU be assessed using large, cross-sectional studies. A consistent approach that allows meaningful comparisons of CAM prevalence across EU member states needs to be based on a clearly defined set of common CAM practices and treatments. A common survey methodology, including standardized questionnaires translated into the various national languages, is essential to ensure the comparability of data. This approach must also be based on the principles of good epidemiological research [14].

### Citizens’ attitudes and needs regarding CAM

Data on citizens’ attitudes and needs regarding CAM are very limited, with information available for fewer than half of the EU member states. Moreover, the methodology and results of previous studies have been poorly reported. Most of the research conducted to date has been drawn from UK samples, and only a few studies have focused specifically on citizens’ needs [8]. The results of these studies indicate that patients often choose CAM because they are unsatisfied with conventional medical treatments [8,9]. Furthermore, compared to other stakeholders in the health sector, patients appear to value different aspects of CAM practice, making their involvement in CAM research crucial. An important aspect of our findings was that the EU public urgently needs more reliable and accessible information about CAM [8,15]. These attitudes and needs have been taken into account and are reflected in the roadmap. In addition, EU citizens’ and patients’ attitudes and needs should be considered in CAM research by disseminating information about CAM and assessing the outcomes of CAM treatment from the patients’ perspective.

A structured approach to CAM research that is relevant to the public’s needs requires comparable data from all EU member states so that studies can be conducted in parallel in several countries whenever possible. Examples of such approaches include:

• large scale surveys based on validated questionnaires;

• qualitative interviews and fieldwork studies with in-depth explorations of local experiences and practice; and

• interdisciplinary research involving mixed-methods with CAM providers and clients as research partners.

### Safety

People take the safety in account when they make decisions about CAM [8]. The considerable differences in the definitions [11], regulation [7] and provision [12] of CAM mean that overarching statements about its safety are inadequate. Furthermore, most CAM treatments have been developed over very long periods and have not undergone robust safety evaluation prior to use [16]. Although safety has also been an important issue among CAM researchers over the past decade [10], it is still poorly understood. A detailed understanding of these issues and the ability to compare treatments based on their risk-benefit ratios are crucial if stakeholders are to make appropriate decisions regarding the use of CAM. In addition, because CAM is often used alongside conventional or other non-conventional medical treatments, special attention must be paid to the potential for treatment interactions.

We suggest addressing CAM safety on a regular basis. Data from clinical trials should be routinely used to investigate the frequency of the more common side effects, since comparisons to adequate control groups help establish risk-benefit ratios. The reporting of serious side effects in single case studies should be fostered. In the long run, developing a European monitoring system for CAM safety may be desirable but would require more thorough information about CAM prevalence and provision.

### Comparative effectiveness research

One of the main strategic questions in CAM research has been whether to prioritize the evaluation of specific effects of single elements of a multi-component treatment (i.e., efficacy) or to investigate the overall treatment effects in more pragmatic clinical settings (i.e., effectiveness) [10]. Patients and providers need to know when CAM is a reasonable choice, as this enables them to make informed decisions in real-world situations. Unfortunately, clinical research to date has often focused on the specific effects of CAM in ideal and standardized clinical situations that are rarely experienced in clinical practice. Therefore, there is a strong need to investigate neglected real-world scenarios in clinical research.

Current trends in conventional medicine also address the limited impact of efficacy studies on decision making in clinical practice [17]. In response, standards for patient-centred outcomes research are currently being developed internationally [18]. The movement in conventional medicine towards more comparative effectiveness research has focused strongly on evaluating different treatment options by including a more heterogeneous patient sample, using real-world treatment protocols that are less standardized focusing on patient-centred outcomes [19,20].

The advantage of comparative effectiveness research is its capacity to evaluate CAM as an optional add-on to conventional treatment, or as an alternative to it. Such research also allows for the evaluation of complex interventions, medium and long-term clinical effects, and cost-effectiveness in comparison to treatment alternatives. Furthermore, comparative effectiveness research calls for stakeholder involvement to help ensure external validity and relevance [21].

Research in real-world settings is the most promising approach to identifying the possible contributions of a variety of modalities, such as CAM, to the health of the EU public. Future research on CAM should emphasize comparative effectiveness analysis as a way to obtain data that are valuable to all stakeholders and provide useful guidance in a pragmatic clinical context.

### Context and meaning effects

There is an on-going debate about the nature, size and relevance of clinical effects in CAM. It has been repeatedly pointed out that CAM interventions seem to be associated often with strong context and meaning effects [10,22]. These so-called non-specific effects sometimes seem to be more powerful than specific effects, such as those elicited by needling a particular point in acupuncture [23]. Therefore, despite small or even non-existent specific effects some CAM interventions seem to be clinically as effective or even more effective than guideline-based conventional treatments [24,25]. The context in which CAM treatments are provided appears to be important to patients, who may choose CAM because of the patient-provider relationship [9] or their beliefs about a treatment [8]. Some methods have been suggested for distinguishing between specific and non-specific effects [10], but most CAM treatments, and indeed most clinical practice, cannot be reduced to a single therapeutic ingredient [26]. In combination, factors such as setting, diagnosis and personal interactions are likely to be responsible in part for the treatment outcome. We therefore see a strong need for research investigating how, and the extent to which, these factors influence outcomes.

Valid and reliable tools are needed to assess components of meaning and context effects, as doing so will facilitate clinical research and allow study results to be compared. Special emphasis should be placed on the question of whether CAM is associated with effects that are different from those in conventional medicine. Understanding the mechanisms behind these effects will help in identifying the appropriate scope and limits of CAM, as well as those of conventional medical treatments. Such research could lead to a better understanding of the mechanisms underlying CAM, clarify the value of CAM for patients and the general public, and help politicians when making reimbursement decisions. Patient and provider expectations, and the time required for diagnosis and treatment, are examples of context and meaning effects that should be included in clinical research on CAM. Researchers should attempt to differentiate these from the intrinsic impact of any specific intervention. However, given the importance of context and meaning effects in all fields of medicine, we believe that research into this area should be a priority in general.

### Health care integration

There are many different CAM modalities and treatments in the EU, as well as a wide variety of ways in which CAM is provided to patients [7,12]. For example, approaches to integrating CAM into health care systems have been described as “opposition”, “integration” and “pluralism” models. In some European countries, CAM is provided as a private service and is often unregulated. In other countries, the education and training of CAM providers are regulated, as is the provision of CAM services [12]. In yet other countries, some CAM treatments are even provided within the public health care system at the national or regional level, and in some cases is reimbursed by public payers. In general, however, the accessibility of CAM remains a major issue for the EU public [8].

The various models of CAM provision in the EU are likely to affect health care in different ways. Provision outside of publicly funded health care allows a free choice of treatments and respects the freedom of individuals within the EU as providers or users of CAM. However, safety and equity of access might be easier to achieve in countries where CAM is provided within the public healthcare system.

At the moment, there is no consensus about the best model for integrating effective CAM treatment, such as acupuncture for pain, into the health care system. Also, it is unlikely that there is one model that can fit all the needs of the different regulatory systems. We recommend evaluating concurrently the various existing models of CAM integration to identify their strengths and limitations. Furthermore, innovative models of CAM provision should be developed to address the needs of the public appropriately.

### Methodological considerations

There is broad consensus in the scientific literature that commonly accepted research methods in conventional medicine can and should also be applied to CAM [10]. Quantitative approaches, such as observational studies and randomized controlled trials, are needed to answer questions about prevalence, safety, effectiveness, efficacy and cost-effectiveness. At the same time, qualitative research is needed to help us understand what really happens in CAM treatments, what outcomes are of interest to the public when choosing CAM, and how CAM practitioners perceive their individual practice. We see qualitative and quantitative methods not as competing, but as complementary.

Potential users of CAM, policy makers, health care providers, and health care payers should be involved at each stage in the development of research questions and study design. Involving stakeholders helps ensure the real-world relevance of CAM research and, when done in a systematic and transparent manner, improves the impact of this research on health care systems. In addition, qualitative methods, such as interviews and focus groups, and quantitative methods, such as surveys, can be useful in assessing stakeholders’ perspectives systematically.

Effective criteria for selecting CAM treatments for future research on prevalence of use, expected impact on clinical practice, and overall feasibility have been proposed in the literature [10] and have already guided research in countries such as the US and Australia [13]. In Europe, such criteria should be developed in a consensual process with stakeholders while taking into account the clinical and economic relevance of any research proposal.

Although we were successful in finding a definition of CAM that fits well into the medical tradition of the EU countries included in the CAMbrella project [11], a valid and comprehensive taxonomy with clear definitions of different CAM procedures still needs to be developed internationally within a transparent consensus process. Until then, CAM researchers must provide study participants and the scientific community with clear descriptions of their understanding of CAM.

### Strategic implications

In order to achieve CAMbrella’s vision, there is need for stronger institutional support of CAM research. So far, Europe has lagged behind North America, Asia and Australia in terms of structural research funding in CAM [13,15]. A lack of institutional support at the European level has resulted in the fragmented picture of CAM described in CAMbrella. Indeed, research funding to date has been driven mainly by particular interests from stakeholders such as patient interest groups or CAM providers. Increased institutional support at the European level is needed to promote research on the topics proposed in the roadmap and to ensure methodological quality.

We recommend first establishing a European CAM research coordination office to foster systematic communication between EU governments, the public, charitable and industry funders, researchers, and other stakeholders. Its aim would be to inform the public about recent developments in CAM research, and to disseminate information about research strategy developments and funding among researchers.

We propose an EU-funded European Centre for CAM (ECCAM) comparable to the National Center for Complementary and Alternative Medicine (NCCAM) at the National Institutes of Health in the US. This depends on the generation of political will at the EU and member state level. The aim of the centre would be to stimulate and support high-quality research on CAM in the EU through an independent research strategy aligned with EU health policy and through its own capacity to fund projects and fellowships. We also recommend improving the quality of CAM research by investing in education, training and collaboration in the CAM research community across Europe and beyond.

## Summary

Although the volume of research into CAM in Europe has increased over the past two decades, there are still insufficient data on the prevalence, effectiveness, efficacy, safety and health economic benefits of most CAM treatments. As a result, patients, providers and other stakeholders are unable to access rigorous and reliable evidence on CAM. We have put forward a roadmap for a strategic research policy to address this deficiency and create a foundation for informed decisions. Our recommendations are based on extensive systematic reviews investigating the current state of CAM in the EU and were shaped in collaboration with key international stakeholders and CAM research experts in a transparent and structured manner.

CAMbrella recommends that we develop:

• reliable knowledge about the provision and use of CAM in the EU, especially with regard to national differences;

• real-world knowledge to help patients and health care providers decide when CAM treatment is appropriate; and

• knowledge about the EU public’s interest in CAM, especially regarding different models of CAM and conventional health care integration, and the implementation of this interest in the research agenda.

CAM research must use generally accepted and appropriate research methods to build a solid evidence base. These include quantitative and qualitative approaches, particularly in mixed-methods studies.

The CAMbrella research roadmap reflects international trends in research [13]. It can be compared to the latest NCCAM strategic plan “Exploring the Science of CAM: Third Plan 2011-2015” [27], whose five main objectives are (1) to advance research on mind and body interventions, practices and disciplines, (2) advance research on CAM natural products, (3) increase understanding of real-world patterns and outcomes of CAM use and its integration into health care and health promotion, (4) to improve the capacity of the field to carry out rigorous research and (5) to develop and disseminate objective, evidence-based information on CAM interventions. Objectives 3 to 5 are comparable to the research focus in CAMbrella, with its strong emphasis on CAM research in real-world settings and the development and improvement of research capacity. Importantly, however, CAMbrella pays special attention to the situation in the EU. The lack of data on CAM prevalence and provision, as well as the diversity of the EU public’s interests in CAM, are central aspects of the CAMbrella roadmap.

The CAMbrella research roadmap identified core areas to evaluate CAM within the EU systematically using generally accepted research methodology, including mixed-methods designs. A robust research strategy depends on the funding of a European CAM coordinating research office and of a European Centre for CAM to realize the CAMbrella research roadmap and enhance clinical knowledge. We are confident that the suggestions put forward in the roadmap can guide CAM research so that it will make a stronger contribution to the needs of the public, health care payers and health care providers in Europe.

## Competing interests

The authors declare that they have no competing interests.

## Authors’ contributions

CAMbrella members FF, GL, CW, KvA, FC, TF, VF, HJ, BR, BU, WW and BB took part in all stages of the consensus process and contributed the results from their respective works within CAMbrella. FF drafted a first version of the manuscript in collaboration with GL, CW and BB. KL was invited expert to the consensus process and contributed a section to the draft. All authors read and corrected the draft and consent to their names on the final manuscript.

## Pre-publication history

The pre-publication history for this paper can be accessed here:

http://www.biomedcentral.com/1472-6882/14/46/prepub

## References

[B1] Eisenberg DM Davis RB Ettner SL Appel S Wilkey S Van Rompay M Kessler RC Trends in alternative medicine use in the United States, 1990-1997: results of a follow-up national survey JAMA 1998 280 1569 1575 10.1001/jama.280.18.1569 9820257

[B2] Härtel U Volger E Use and acceptance of classical natural and alternative medicine in Germany–findings of a representative population-based survey Forsch Komplementarmed 2004 11 327 334 10.1159/000082814 15604623

[B3] Bücker B Groenewold M Schoefer Y Schäfer T The use of complementary alternative medicine (CAM) in 1 001 German adults: results of a population-based telephone survey Gesundheitswesen 2008 70 e29 e36 10.1055/s-2008-1081505 18785094

[B4] Bodeker G Ong CK Grundy C Burford G Shein K WHO Global Atlas of Traditional, Complementary and Alternative Medicine 2005 Kobe: World Health Organization

[B5] Committee on the Use of Complementary and Alternative Medicine by the American Public Complementary and Alternative Medicine in the United States 2005 Washington, DC: National Academies Press (US) 22379647

[B6] Weidenhammer W Lewith G Falkenberg T Fønnebø V Johannessen H Reiter B Uehleke B von Ammon K Baumhöfener F Brinkhaus B EU FP7 project “CAMbrella” to build European research network for complementary and alternative medicine Forsch Komplementarmed 2011 18 69 76 10.1159/000327310 21576975

[B7] Wiesener S Falkenberg T Hegyi G Hök J Roberti di Sarsina P Fønnebø V Legal status and regulation of complementary and alternative medicine in Europe Forsch Komplementarmed 2012 19 29 36 10.1159/000343125 23883942

[B8] Nissen N Schunder-Tatzber S Weidenhammer W Johannessen H What attitudes and needs do citizens in europe have in relation to complementary and alternative medicine? Forsch Komplementarmed 2012 19 9 17 10.1159/000342710 23883940

[B9] Eardley S Bishop FL Prescott P Cardini F Brinkhaus B Santos-Rey K Vas J von Ammon K Hegyi G Dragan S Uehleke B Fønnebø V Lewith G A systematic literature review of complementary and alternative medicine prevalence in EU Forsch Komplementarmed 2012 19 18 28 10.1159/000342708 23883941

[B10] Fischer HF Junne F Witt C von Ammon K Cardini F Fønnebø V Johannessen H Lewith G Uehleke B Weidenhammer W Brinkhaus B Key issues in clinical and epidemiological research in complementary and alternative medicine - a systematic literature review Forsch Komplementarmed 2012 19 51 60 10.1159/000343126 23883945

[B11] Falkenberg T Lewith G Roberti di Sarsina P von Ammon K Santos-Rey K Hök J Frei-Erb M Vas J Saller R Uehleke B Towards a Pan-European definition of complementary and alternative medicine - a realistic ambition? Forsch Komplementarmed 2012 19 6 8 10.1159/000343812 23883939

[B12] Von Ammon K Frei-Erb M Cardini F Daig U Dragan S Hegyi G Roberti di Sarsina P Sörensen J Lewith G Complementary and alternative medicine provision in Europe - first results approaching reality in an unclear field of practices Forsch Komplementarmed 2012 19 37 43 10.1159/000343129 23883943

[B13] Hök J Lewith G Weidenhammer W Santos-Rey K Fønnebø V Wiesener S Falkenberg T International development of traditional medicine / complementary and alternative medicine research - What can Europe learn? Forsch Komplementarmed 2012 19 44 50 10.1159/000342724 23883944

[B14] Von EE Altman D Egger M Pocock S Gotzsche P Vandenbroucke J Strobe Initiative: The Strengthening the Reporting of Observational Studies in Epidemiology (STROBE) statement: guidelines for reporting observational studies Ann Intern Med 2007 147 573 578 10.7326/0003-4819-147-8-200710160-00010 17938396

[B15] Reiter B Baumhöfener F Dlaboha M Odde Madsen J Regenfelder S Weidenhammer W Building a sustainable complementary and alternative medicine research network in Europe Forsch Komplementarmed 2012 19 61 68 10.1159/000342723 23883946

[B16] Fonnebo V Grimsgaard S Walach H Ritenbaugh C Norheim AJ MacPherson H Lewith G Launsø L Koithan M Falkenberg T Boon H Aickin M Researching complementary and alternative treatments–the gatekeepers are not at home BMC Med Res Methodol 2007 7 7 10.1186/1471-2288-7-7 17291355 PMC1800863

[B17] Tunis S Stryer D Clancy C Practical clinical trials JAMA 2003 290 1624 1632 14506122 10.1001/jama.290.12.1624

[B18] Our Questions, Our Decisions: Standards for Patient-centered Outcomes Research http://pcori.org/assets/MethodologyReport-Comment.pdf

[B19] VanLare JM Conway PH Sox HC Five next steps for a new national program for comparative-effectiveness research N Engl J Med 2010 362 970 973 10.1056/NEJMp1000096 20164480

[B20] Craig P Dieppe P Macintyre S Michie S Nazareth I Petticrew M Developing and evaluating complex interventions: the new Medical Research Council guidance Br Med J 2008 337 979 983 10.1136/bmj.a979

[B21] Hoffman A Montgomery R Aubry W Tunis SR How best to engage patients, doctors, and other stakeholders in designing comparative effectiveness studies Health Aff 2010 29 1834 1841 10.1377/hlthaff.2010.0675 20921483

[B22] Kaptchuk TJ The Placebo effect in alternative medicine: can the performance of a healing ritual have clinical significance? Ann Intern Med 2002 136 817 825 10.7326/0003-4819-136-11-200206040-00011 12044130

[B23] Linde K Niemann K Schneider A Meissner K How large are the nonspecific effects of acupuncture? A meta-analysis of randomized controlled trials BMC Med 2010 8 75 10.1186/1741-7015-8-75 21092261 PMC3001416

[B24] Haake M Muller H Schade-Brittinger C Basler H Schafer H Maier C Endres H Trampisch H Molsberger A German Acupuncture Trials (GERAC) for chronic low back pain: randomized, multicenter, blinded, parallel-group trial with 3 groups Arch Intern Med 2007 167 1892 1898 10.1001/Archinte.167.17.1892 17893311

[B25] Linde K Allais G Brinkhaus B Manheimer E Vickers A White AR Acupuncture for migraine prophylaxis Cochrane Database Syst Rev 2009 1 CD001218 19160193 10.1002/14651858.CD001218.pub2 PMC3099267

[B26] Paterson C Dieppe P Characteristic and incidental (placebo) effects in complex interventions such as acupuncture Br Med J 2005 330 1202 1205 10.1136/bmj.330.7501.1202 15905259 PMC558023

[B27] NCCAM Strategic Plan 2011-2015 http://nccam.nih.gov/about/plans/2011

